# S87. THE INITIAL CHANGE IN THE SERUM LEVEL OF C-REACTIVE PROTEIN IN ACUTE PSYCHOSIS IS ASSOCIATED WITH COGNITIVE PERFORMANCE IN LATER PHASES

**DOI:** 10.1093/schbul/sby018.874

**Published:** 2018-04-01

**Authors:** Farivar Fathian, Else-Marie Løberg, Rolf Gjestad, Vidar Martin Steen, Rune Kroken, Hugo A Jørgensen, Erik Johnsen

**Affiliations:** 1 NKS Olaviken Gerontopsychiatric Hospital; 2 University of Bergen, Haukeland University Hospital; 3 Haukeland University Hospital; 4 University of Bergen

## Abstract

**Background:**

Inflammatory processes have been implicated in the pathophysiology of schizophrenia and related psychosis and could be particularly relevant to the associated cognitive deficits. The C-reactive protein (CRP) serves as a general marker of inflammation, and inverse relationships between CRP levels and cognitive performance in acute psychosis have been demonstrated. Here we aimed to investigate how the serum level and initial change of CRP in acutely admitted patients with psychosis were correlated with cognitive performance during a 6-months follow-up period.

**Methods:**

The study is part of a pragmatic, randomized trial comparing four different second-generation antipsychotic drugs, and consists of 208 acute phase patients recruited at admittance for psychosis (schizophrenia, schizoaffective disorder, acute and transient psychotic disorder, delusional disorder, drug-induced psychosis, bipolar disorder except for manic psychosis, or major depressive disorder with psychotic features). The present study reports data for all treatment groups collectively, and does not compare treatment groups.

Measurements of CRP and cognitive performance were conducted at baseline (T1) and after an average of 4 weeks after inclusion (T2). Cognition was assessed after 3 months (T3) and 6 months (T4) of follow-up.

**Results:**

Global cognition improved during the follow-up period of 6 months, especially in the T1–T2 interval. The different cognitive subdomains showed different time-dependent profiles of improvement, with memory and attention improving significantly also in the later phases. Reduction of the CRP level during the initial follow-up interval (T1–T2) was associated with increased overall cognitive performance in the T2–T4 interval, but not in the T1–T2 interval. For the cognitive subdomains, we found an inverse association between change in CRP level and learning (T1–T2 interval), verbal abilities (T2–T4 interval), and attention (T2–T3 interval).

**Discussion:**

The main finding of the present study was that the global cognitive performance continued to improve from the initial phase (baseline to 4 weeks) of acute psychosis to the later phase (4 weeks–6 months), and was predicted by the change in CRP level that was observed during the initial phase (baseline–4 weeks) of the treatment. These findings might indicate a prolonged effect of inflammatory processes on cognition in acute psychosis, stretching beyond the initial phase.

There is substantial evidence that inflammatory processes are involved in the cognitive performance in psychosis. CRP levels have been associated with cognitive impairment in patients with schizophrenia.

**Conclusions:**

These findings indicate that initial changes in the serum level of CRP in the acute phase of psychosis may predict cognitive functioning in later phases of the disease.

